# *Wolbachia* significantly impacts the vector competence of *Aedes aegypti* for Mayaro virus

**DOI:** 10.1038/s41598-018-25236-8

**Published:** 2018-05-02

**Authors:** Thiago Nunes Pereira, Marcele Neves Rocha, Pedro Henrique Ferreira Sucupira, Fabiano Duarte Carvalho, Luciano Andrade Moreira

**Affiliations:** Centro de Pesquisas René Rachou- Fiocruz, Endossimbiontes e Interação Patógeno-Vetor, Belo Horizonte, Minas Gerais, 30190-002 Brazil

## Abstract

*Wolbachia*, an intracellular endosymbiont present in up to 70% of all insect species, has been suggested as a sustainable strategy for the control of arboviruses such as Dengue, Zika and Chikungunya. As Mayaro virus outbreaks have also been reported in Latin American countries, the objective of this study was to evaluate the vector competence of Brazilian field-collected *Ae*. *aegypti* and the impact of *Wolbachia* (*w*Mel strain) upon this virus. Our *in vitro* studies with Aag2 cells showed that Mayaro virus can rapidly multiply, whereas in *w*Mel-infected Aag2 cells, viral growth was significantly impaired. In addition, C6/36 cells seem to have alterations when infected by Mayaro virus. *In vivo* experiments showed that field-collected *Ae*. *aegypti* mosquitoes are highly permissive to Mayaro virus infection, and high viral prevalence was observed in the saliva. On the other hand, *Wolbachia*-harboring mosquitoes showed significantly impaired capability to transmit Mayaro virus. Our results suggest that the use of *Wolbachia*-harboring mosquitoes may represent an effective mechanism for the reduction of Mayaro virus transmission throughout Latin America.

## Introduction

Mosquitoes are effective at rapidly disseminating arboviral diseases for which outbreaks are common throughout the world. The mosquito *Aedes aegypti* is a species with nearly world-wide distribution that is highly anthropophilic and extremely opportunistic^1,2^. This insect vector severely increases the economic burden to public health, since they are involved in the transmission of disease agents such as Zika (ZIKV), Dengue (DENV), Chikungunya (CHIKV) and Yellow Fever (YFV)^3–5^ as well as others such as Mayaro virus (MAYV). MAYV is a member of the *Togaviridae* family in the genus *Alphavirus* and its transmission cycle involves mainly *Haemagogus* mosquitoes. However, laboratory studies have shown that *Aedes* spp. can be competent vectors for this virus^6,7^. MAYV was first identified in humans in 1954 in Mayaro, Trinidad and has subsequently been found in French Guiana, Suriname, Venezuela, Louisiana, Peru, Bolivia, and Brazil^6,8–14^. Sporadic cases of MAYV have been reported regularly across Brazil, including the Northern, Northeastern and Central West regions, with frequent occurrence in the states of Pará, Amazonas, Acre, and Mato Grosso^11,13,15–18^. MAYV infection symptoms are similar to those of DENV and CHIKV and are characterized by frontal headache, high fever, epigastric pain, myalgia, chronic arthralgia (more associated with CHIKV), maculopapular eruption, photophobia, and nausea. These symptoms may persist for several months, and can be incapacitating for infected persons^19^.

In recent years, several methods have been proposed for controlling arboviruses, including the use of *Wolbachia* as a biological control agent. This bacterium does not naturally occur in *Ae*. *aegypti*, but when introduced into this vector, has the ability to greatly reduce its capacity to harbor and transmit pathogens^20–28^. *Wolbachia* has been used in some parts of the world as a tool to control the transmission of DENV and other arboviruses (http://www.eliminatedengue.com/program). Mosquitoes harboring *Wolbachia* are released in the wild, and after establishment of the bacteria into native populations, pathogen infection and transmission can be reduced. More than five years after the introduction of *Wolbachia*-infected mosquitoes to natural populations in Australia, almost 100% of the *Ae*. *aegypti* populations still host the bacteria and have maintained the ability to block DENV^29,30^.

Taking into consideration the strength of *Wolbachia* as tool to reduce arboviral transmission and the relevance of MAYV as a potential human pathogen, it is important to explore the effect of this bacterium towards this virus. Here, we determined the vector competence of *Ae*. *aegypti* and the efficiency of *Wolbachia* (*w*Mel strain) against MAYV infection as an alternative strategy to control the transmission of this virus.

## Results

### Morphological alterations in C6/36 cells caused by MAYV

**Figure 1:**
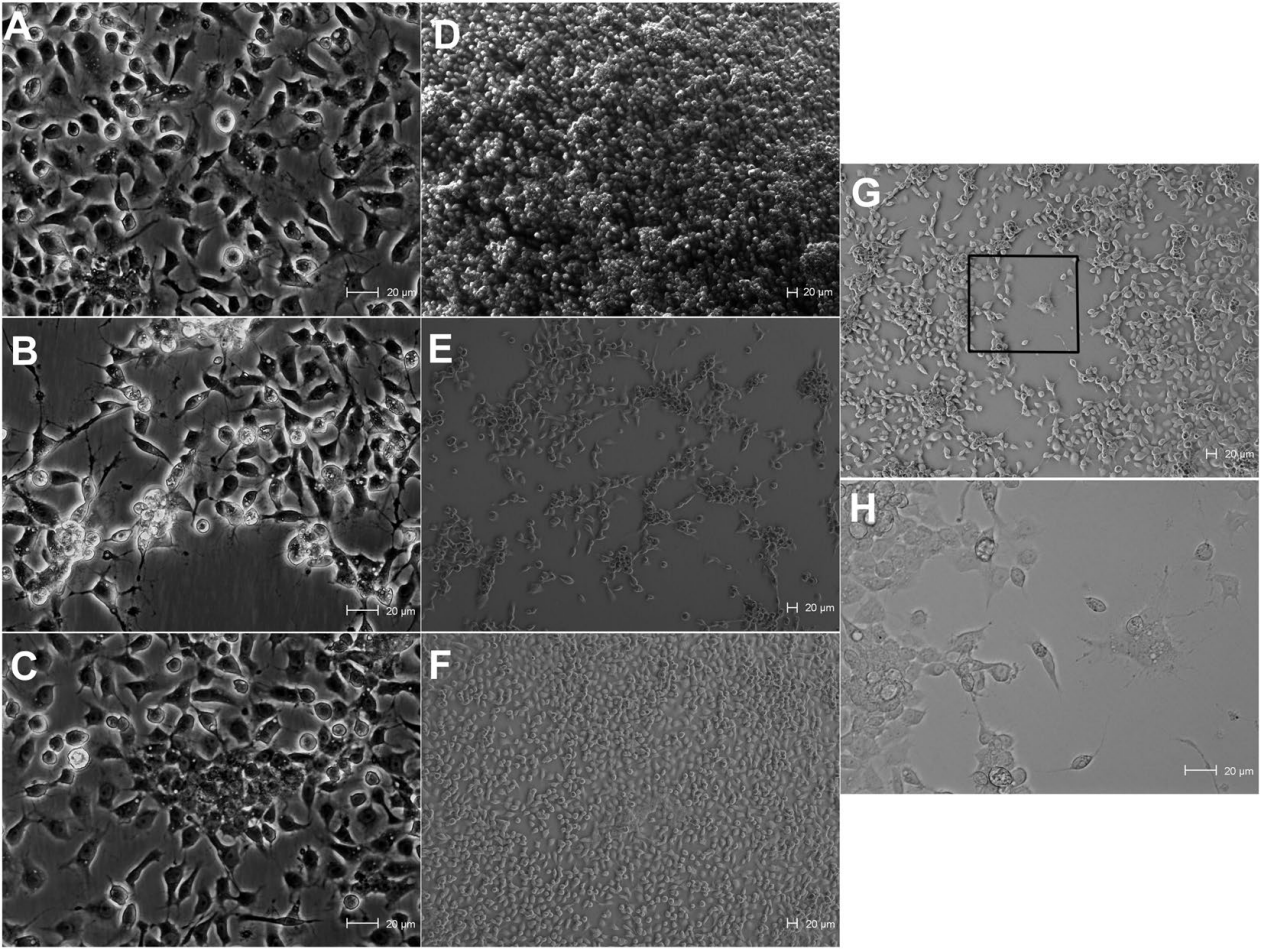
Optical microscopy of *Ae*. *albopictus* (C6/36) cell cultures infected by Mayaro or DENV. Uninfected C6/36 cells (**A**), MAYV-infected C6/36 cells (**B**), DENV-1-infected C6/36 cells (**C**). Cells were cultured in flasks and evaluated directly under optical microscopy without any preparation. These images were taken at 4 days post-infection (original magnification = 320x). Uninfected C6/36 cells (**D**), MAYV-infected C6/36 cells (**E**), DENV-1-infected C6/36 cells (**F**), these images were taken at 6 days post-infection (magnification = 100x). MAYV-infected C6/36 cells at 5 days post-infection (magnification = 100x) (**G**) the rare cytopathic effect caused by MAYV and (**H**) highlight for the cytopathic effect (magnification = 320x).

In order to verify whether MAYV would cause morphological alterations in C6/36 cells, we cultured MAYV and DENV-infected and uninfected cells (Fig. 1). MAYV seems to have caused some cell alterations, as well as a decrease in number of cells and a faster viral replication when compared to DENV. In addition, in this experiment an uncommonly cytopathic effect was observed only once time when MAYV was presented, which is similar what is caused by DENV, i.e. multinuclear giant cell formation due to the fusions of cytoplasmic membranes (Fig. 1G). We also observed a decreasing number of monolayer cells over time in the cells infected by MAYV (Fig. 1B and E). Cells were cultured uninfected (Fig. 1A and D) and infected DENV serotype 1 (Fig. 1C and F) to compare observed events.

### *In vitro* viral replication and *Wolbachia* blocking effect

**Figure 2:**
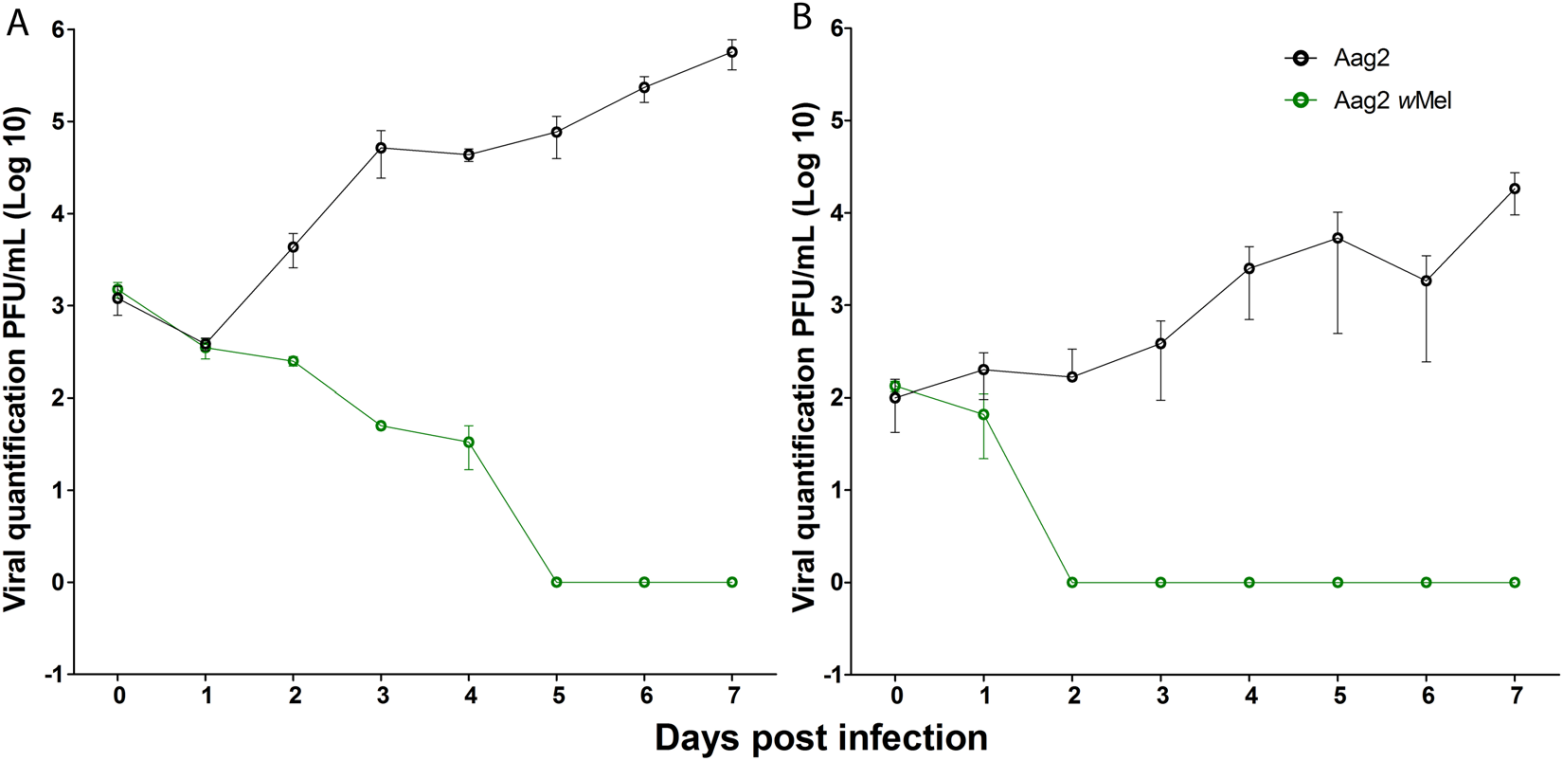
Kinetics of MAYV viral growth and the *Wolbachia* blocking effect. (**A**,**B**) Aag2 cells were challenged with two different MOIs: (**A**) MOI 0.1 and (**B**) MOI 0.01. Aag2 without *Wolbachia* (black line) maintained steady growth for both MOIs. The Aag2-*w*Mel cell line (green line) had significant effect on MAYV growth. MOI of 0.1 exhibited a later blocking effect. Viral titration of MAYV-containing supernatant was determined by plaque assay for 3 days after infection in Vero cells. Cells were infected in triplicate, and the values represent means ± SD.

In order to check whether *Wolbachia* would exhibit any effect towards MAYV we firstly performed *in vitro* tests using *Ae*. *aegypti* cells (Aag2 with and without *Wolbachia*). Unfortunately, we did not have the C6/36 cells (also with *Wolbachia*) which is widely used for virus replication, to perform these experiments. *In vitro* tests in Aag2 cells showed that the kinetics of MAYV growth had a direct correlation with the different MOIs. Both MOIs in Aag2 cells without *Wolbachia* showed a similar growth pattern for MAYV (Fig. 2A and B). However, the MOI 0.1 produced higher viral titers compared to MOI 0.01. In the Aag2-*w*Mel cells, we observed more rapid viral inhibition at MOI 0.01. The MOI 0.01 blocking effect started on the second day and was maintained until the end of the experiment (Fig. 2A). For the MOI 0.1, the blocking effect started between the third and fifth day and remained constant until the sixth day (Fig. 2B).

### MAYV infection in mosquitoes

To evaluate mosquito competence for MAYV, we processed the head + thorax of individual mosquitoes at different time points post-infection. Among the 55 Br (control –*Wolbachia* uninfected) mosquitoes fed with fresh virus supernatant (MAYV), 49 (89.09%) had an infection level of >10^3^ viral copies, 5 (9.09%) had an infection level of <10^3^, and only 1 (1.82%) was uninfected. The median viral load of positive samples was 1.88 × 10^4^_,_ 1.65 × 10^7^ and 5.13 × 10^6^ copies/head + thorax for 4, 14 and 28 dpi, respectively.

**Figure 3:**
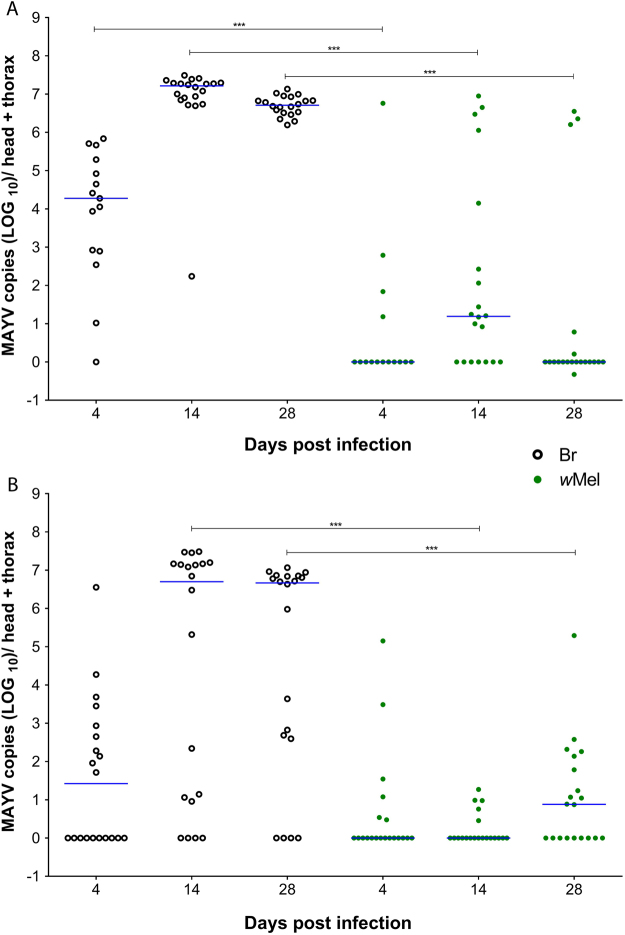
Susceptibility of *Aedes aegypti* and the ability of *Wolbachia* to block MAYV. (**A**,**B**) Mosquitoes were orally challenged with either (**A**) fresh virus or (**B**) frozen virus samples. Br mosquitoes (black circle) and *w*Mel *Wolbachia* (green circle). Each circle represents a single adult female, and the blue lines indicate the median number of MAYV copies in each treatment. ^∗∗∗^*P* < *0*.*0001*; Mann-Whitney U test.

For the *w*Mel samples (*Wolbachia*-infected), among 55 mosquitoes, 34 (61.82%) were uninfected and of the remaining 21 mosquitoes, 12 (21.82%) had an infection level of <10^3^, and 9 (16.36%) had an infection level of >10^3^ viral copies. The median of the infected samples was 0, 1.55 × 10^1^ and 0 copies/head + thorax for 4, 14 and 28 dpi (Fig. 3A). The Mann-Whitney test showed a significant difference between the groups (Br and *w*Mel): *P* = *0*.*0003* for 4 dpi, *P* < *0*.*0001* for 14 dpi and *P* < *0*.*0001* for 28 dpi. The prevalence of MAYV infection for fresh virus was significantly reduced among *Wolbachia*-infected mosquitoes (Fisher’s exact test, *P* < 0.0001; Table 1). The infection prevalence (head + thorax) in the Br and *w*Mel groups was 93.33% and 26.67% at 4 dpi (*P* = 0.005), 100% and 65% (*P* = 0.083) at 14 dpi and 100% and 25% (*P* < 0.0001) at 28 dpi, respectively.

**Table 1:** *Aedes* *aegypti* were orally infected with fresh and frozen MAYV.

MAYV	MAYV Titer (PFU/mL)	Days Post-infection	*w*Mel	Br
			Head/Thorax Infection Rate	
Fresh virus	>10^9^	4	26.67(4/15)	93.33 (14/15)
		14	65 (13/20)	100 (20/20)
		28	25 (5/20)	100 (20/20)
Frozen virus	>10^8^	4	30 (6/20)	50 (10/20)
		14	25 (5/20)	80 (16/20)
		28	55 (11/20)	80 (16/20)

Infection rates are given as percentages. n = 15 or 20 per group unless specified; PFU, plaque-forming units; *w*Mel: *Wolbachia*-infected; Br: *Wolbachia*-uninfected.

In the experiment where frozen virus was used, out of 60 BR mosquitoes, 29 (48.33%) had an infection level of >10^3^ viral copies, 13 (21.67%) had an infection level of <10^3^, and 18 (30%) were uninfected. The median of the infected samples was 2.59 × 10^1^, 5.01 × 10^6^ and 4.66 × 10^6^ for 4, 14 and 28 dpi, respectively. Regarding the *w*Mel samples, 38 (63.33%) out of the 60 mosquitoes were negative and of the remaining 22 mosquitoes, 19 (31.67%) had an infection level of <10^3^, and 3 (5%) had an infection level of >10^3^ viral copies (Fig. 3B). The median of the infected samples was 0, 5.5 × 10^1^ and 0 for 4, 14 and 28 dpi, respectively. The Mann-Whitney U test showed a significant difference between the two groups (Br and *w*Mel) at 14 dpi (*P* <0.0001) and 28 dpi (*P*< 0.0002), but not at 4 dpi (*P* = 0.1022). The infection prevalence for frozen MAYV was significantly different among *Wolbachia*-infected mosquitoes at 14 dpi (Fisher’s exact test, *P* < 0.0001) (Table 1). The infection prevalence for head + thorax in Br and *w*Mel was 50% and 30% at 4 dpi (*P* = 0.3332), 80% and 25% (*P* = 0.0104) at 14 dpi, and 80% and 55% (*P* = 0.1760) at 28 dpi, respectively.

In order to check whether *Wolbachia* density would have influence on the amount of virus in mosquitoes, we have selected samples from both experiments that showed higher levels of virus in mosquito tissues as well as MAYV-negative samples. Our results show no significant difference of *Wolbachia* density between the two groups (Mann-Whitney U test, P = 0.6349). (Supplementary Figure S1).

### Saliva injection and MAYV load in saliva

**Figure 4:**
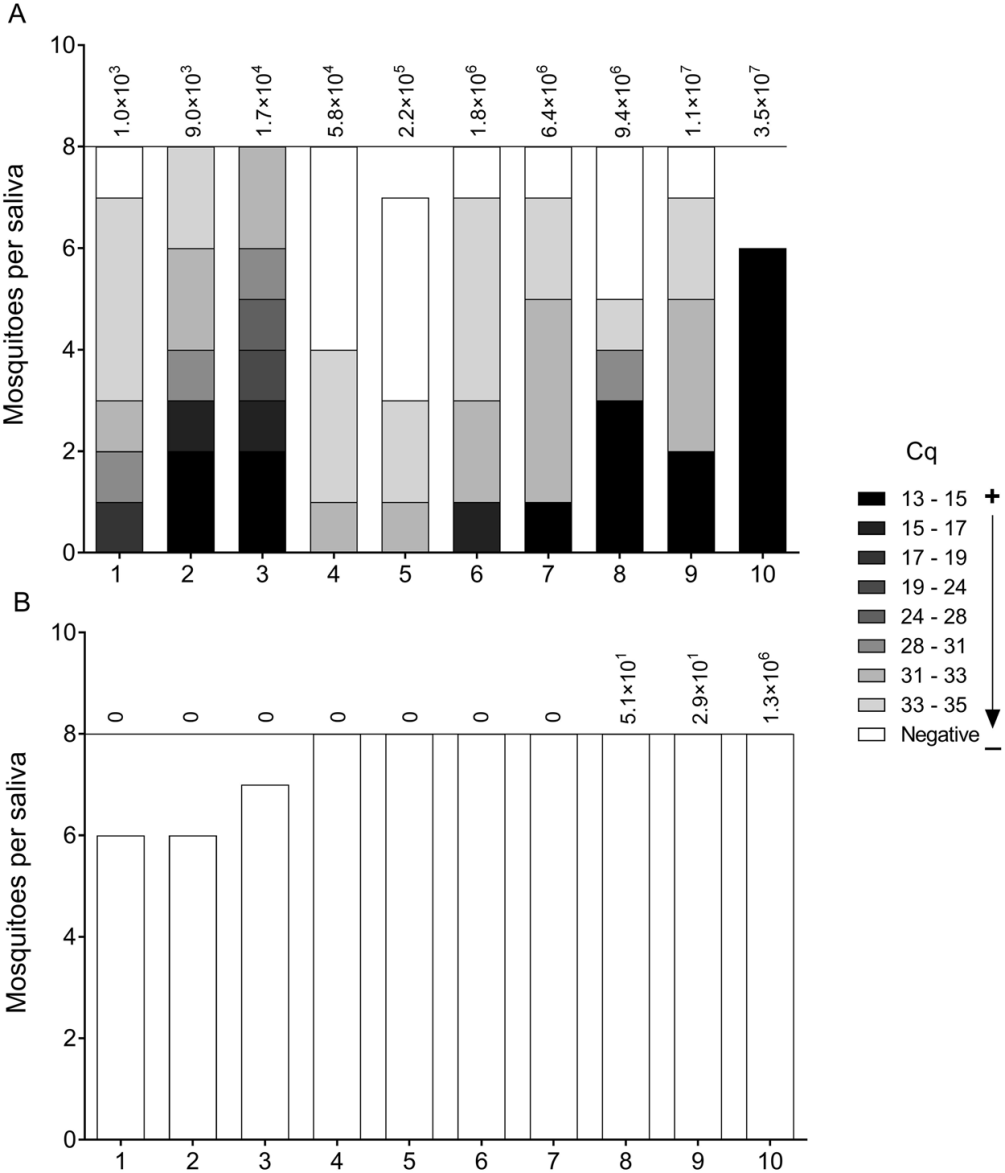
Injection of saliva into naive mosquitoes. Saliva was collected from Br and *w*Mel mosquitoes infected with fresh virus at 7 dpi. All of the Br saliva samples (**A**) were infectious, but no infections were observed when saliva samples originated from *w*Mel mosquitoes (**B**). The color gradient indicates the infection level and varies according to the quantification cycle (Cq). The most infected are shown in black and there is a color gradient toward the uninfected in white. Values at the top of the graphs show the MAYV copy numbers in the head and thorax of the mosquito that the saliva was collected from (as determined by RT-qPCR).

To verify whether infected mosquitoes were able to transmit the virus, we collected saliva from MAYV infected (*Wolbachia*-positive and negative) mosquitoes and injected it into naive Br mosquitoes. Of the 77 mosquitoes injected with Br saliva (Fig. 4A), 63 (81.81%) became infected with MAYV. In contrast, not a single mosquito out of the 75 injected with saliva from *w*Mel-infected mosquitoes was positive for MAYV (Fig. 4B).

**Figure 5:**
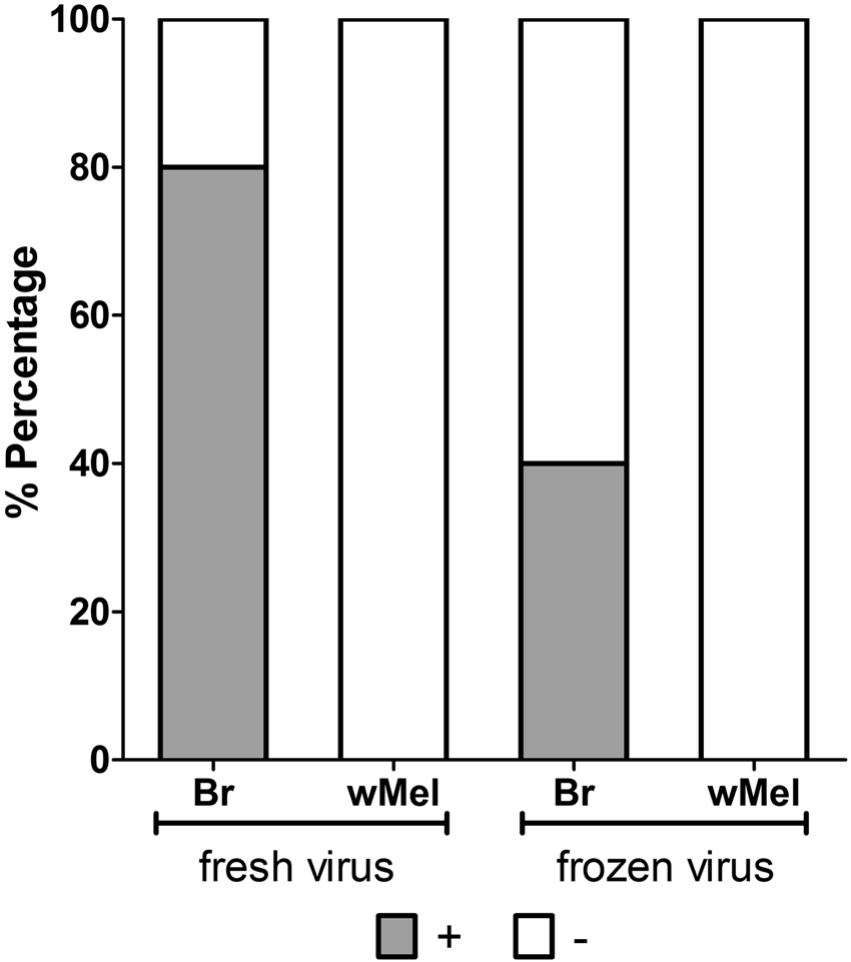
Quantification of MAYV directly from mosquito saliva through RT-PCR at 28 dpi. It was only possible to detect virus in Br mosquitoes (fresh and frozen). However, the numbers virus copies were lower when using frozen virus. It was not possible to detect virus in *w*Mel mosquito saliva. The Fisher’s exact test showed no significant differences between fresh and frozen virus for Br mosquitoes (*P* = *0*.*1698*).

Additionally, we tried to detect MAYV directly in the saliva of both Br and *w*Mel mosquitoes collected at 28 dpi after oral infection with either fresh or frozen virus (Fig. 5). We observed that 8/10 (80%) saliva samples from Br mosquitoes fed on fresh virus were positive, while 4/10 (40%) of the samples from Br mosquitoes fed on frozen MAYV had detectable virus. No MAYV was observed in the 20 *w*Mel saliva samples tested (10 with fresh virus, 10 with frozen samples).

## Discussion

Our results indicate that C6/36 cells infected with MAYV may suffer some alterations such as reduction of cell numbers and an uncommonly cytopathic effect that seems to be limited and rare, promoting faster viral replication compared to DENV-1. In a previous report, the same pattern of growth (fast viral replication) was observed, but no cytopathic effects were reported^31^.

*Ae*. *aegypti* cells (Aag2) can efficiently sustain the growth of MAYV, exhibiting constant viral replication. The replication kinetics of MAYV in Aag2 were quite rapid. *Wolbachia* (*w*Mel)-containing Aag2 cells seem to block MAYV regardless of the MOI. To our knowledge, this is the first report using Aag2 cells for MAYV growth and to evaluate the efficiency of *Wolbachia* against this virus. A previous report used C6/36 cells infected with another *Wolbachia* strain (*w*MelPop) and showed significant reduction of DENV virus replication when compared to *Wolbachia*-uninfected controls^32^.

We observed that Brazilian field populations (Br) were highly permissible to MAYV. Br mosquitoes showed a greater number of viral particles at 14 dpi, with a median of 1.65 × 10^7^ for fresh virus and 5.01 × 10^6^ for frozen virus. *Ae*. *aegypti* is a competent vector for MAYV, as is *Aedes albopictus* and *Aedes scapularis*^6,33^. The percentage of *Ae*. *aegypti* infected with MAYV in the laboratory increased with dosage above a certain threshold^7^. In addition to laboratory studies, Brazilian *Ae*. *aegypti* and *Culex quinquefasciatus* populations have been found to be infected with MAYV in their natural habitats^34^. The transmission of MAYV in an urban cycle has been proposed in Manaus^13^ and Cuiabá^18^. The rapid viral replication (both *in vitro* and *in vivo*) shown here combined with the global distribution of *Ae*. *aegypti*^35^ indicates that this virus may spread through different areas of the world in a short period of time.

*Ae*. *aegypti* mosquitoes harboring *Wolbachia* (*w*Mel) showed a drastic reduction in MAYV infection. Previous studies have shown the ability of different *Wolbachia* strains to block pathogens and reduce the ability of mosquitoes to transmit viruses such as ZIKV, DENV, CHIKV, and YFV, as well as malaria parasites^20,21,23–27,36–39^. Furthermore, several different strains of *Wolbachia* bacterium can cause inhibition, for example the *w*MelPop which is able to block different DENV serotypes as well as other arboviruses^20,27^. Other *Wolbachia* infections, particularly *w*AlbB, *w*Mel and *w*MelPop-CLA, into *Ae*. *aegypti* has been shown to significantly reduce the vector competence of this mosquito in the laboratory^21,24,40^. The list of pathogens that *Wolbachia* exerts an effect upon may possibly be extended as further studies become available.

To determine the transmission of MAYV, saliva originating from Br mosquitoes was injected into naive Br mosquitoes and resulted in high infection rates, confirming that *Ae*. *aegypti* are potential vectors of MAYV. A previous study has shown that MAYV was efficiently transmitted by *Ae*. *aegypti* to suckling mice, showing its potential as a vector for this arbovirus^33^. In contrast, *Wolbachia* significantly inhibited MAYV transmission in *Ae*. *aegypti*. When *w*Mel-mosquito saliva was injected into naive Br mosquitoes, not one of the 75 injected mosquitoes became infected. The same methodology was previously used for ZIKV, and no mosquitoes injected with *w*Mel-originated saliva became infected^26^. Our data show that in addition to becoming infected, *Ae*. *aegypti* mosquitoes can also transmit MAYV; moreover, we show that *Wolbachia* has a strong impact on the transmission of MAYV.

The use of frozen supernatant was shown to limit viral infection in mosquitoes and produced a lower rate of detectable viral particles in saliva. Infection rates and vector competence can be significantly lower for mosquitoes fed with frozen virus^41,42^. In addition, experiments showed that freezing and thawing ZIKV significantly impaired mosquito infection^43^. Therefore, we believe that the use of fresh virus should be the preferred choice, as it can better simulate natural conditions.

Overall, the results presented here suggest that if *Ae*. *aegypti* becomes a vector of MAYV in urban areas, the *w*Mel strain may be used to reduce the prevalence and severity of this arbovirus. Ongoing field trials of *Ae*. *aegypti* mosquitoes harboring *w*Mel are already in place in several countries as part of a global initiative.

## Materials and Methods

### Cell culture

C6/36 *Aedes albopictus* cells were maintained in Leibowitz L-15 medium supplemented with 10% fetal bovine serum (Gibco) and maintained at 28 °C, whereas the Aag2 cells (*Ae*. *aegypti* cell line) were grown on Schneider’s insect medium with L-glutamine (Gibco) supplemented with 10% fetal bovine serum (Gibco) at 28 °C as previously described by Hamel^44^.

### Virus culture

MAYV and DENV stocks were maintained on the C6/36 *Aedes albopictus* cell line previously described by Hamel^44^. The C6/36 cells were grown in adherent flasks (25 cm^2^) to produce large quantities of infected supernatant.

The MAYV was part of a virus collection of the Federal University of Rio de Janeiro and DENV serotype 1 (DENV-1) was isolated during an outbreak in 2015 in Contagem, MG, Brazil.

### Mosquito rearing

Two *Ae*. *aegypti* mosquito lines were used: the F_2_ generation of a (Br) Brazilian field population (*Wolbachia*-uninfected) collected from ovitraps in the suburb of Urca, RJ, Brazil in the beginning of 2017, and mosquitoes harboring the *Wolbachia* strain (*w*Mel) backcrossed with field-collected male mosquitoes from suburb of Urca, RJ, Brazil every five generations to maintain a similar genetic background between the two lines. The methodology used to homogenize the genetic background of the mosquito lines was the same shown by Dutra^45^.

The insects were reared under a 12:12 h photoperiod at 28 °C ± 2 °C with a relative humidity of 60 ± 10%. Larvae were grown in plastic trays containing 300 larvae in 3 liters of water and fed with ½ ground Tetramin tropical fish food tablet each day. Sucrose solution (10%) was continuously provided to adults as a sugar source for feeding.

### Assays

Morphological alterations in MAYV-infected C6/36 cells

Cellular morphology was compared between MAYV-infected and uninfected C6/36 cells with a parallel infection with DENV-1 used as a point of comparison. Three different groups were grown in flasks (25 cm^2^) and maintained under the same conditions: C6/36 only, C6/36 + MAYV, and C6/36 + DENV-1. Cell growth was observed under light microscopy and photographed every day for 7 days.

### *In vitro* viral replication and the *Wolbachia* blocking effect

For this experiment, we used an uninfected cell line and a line in which the *w*Mel *Wolbachia* strain had previously been stably introduced (Aag2- *w*Mel cell line). The *in vitro* blocking assay was performed in a 96-well plate containing 2 × 10^5^ cells per well. The multiplicities of infection (MOIs) tested were 0.1 and 0.01. The viral replication kinetics were examined by collecting supernatant from cells daily up to 7 days.

The supernatant was then frozen at −80 °C and used to infect Vero cells in a semi-solid medium using the carboxymethylcellulose system^46^. The total plaque forming units per milliliter (PFU/mL) were counted three days after the viral infection of the cells. This experiment was repeated three times.

### MAYV mosquito infection

Five-day-old adult female mosquitoes (Br and *w*Mel) were starved for 24 hours prior to oral infection. A mixture of 2:1 virus/blood was offered through glass feeders using pig intestine as the membrane and a water jacket system with the temperature maintained at 37 °C. Mosquitoes were allowed to feed on the blood-virus mixture for 30–60 minutes. Immediately after feeding, fully engorged females were screened and maintained on 10% sucrose for the duration of the experiment.

Mosquitoes were collected from both groups on different days post-infection and stored at −80 °C before processing. In the first experiment, we used fresh supernatant from infected C6/36 cells harvested five days after viral adsorption with a viral titer of >10^9^ PFU/mL. In the second experiment, we used frozen supernatant from infected C6/36 cells with a corresponding viral titer of >10^8^ PFU/mL.

The most important region for virus transmission in the mosquito is the head, where the salivary glands are located^47^; thus, in this experiment, we used only mosquito heads and thoraces. To facilitate analysis, we categorized the number of viral copies found in mosquito head + thorax into 3 groups: those with no viral copies (0), those with less than 1,000 viral copies (<10^3^), and those with more than 1,000 viral copies (>10^3^).

The human blood used in these experiments was obtained as an expired component from a blood bank (Hemominas), and was donated to our group for research purposes, according to the terms of an agreement with René Rachou Institute (OF.GPO/CCO - Nr 224/16).

### Saliva collection and injection

Individual mosquito saliva samples were collected at 7 days post-infection with MAYV. Mosquitoes were anesthetized with CO_2_ and kept on an ice plate while the legs and wings were removed. Each mosquito proboscis was inserted into a 10 µL pipette tip containing a 1:1 solution of 5 µL of sterile fetal bovine serum and 30% sucrose solution. After 30 minutes, the contents of the tips were collected in 0.6 mL tubes and stored at −80 °C until processing. RNA from all samples was extracted using the High Pure Viral Nucleic Acid Kit (Roche) following the manufacturer’s instructions.

Ten undiluted saliva samples from each group (Br and *w*Mel) collected at 7 dpi were injected into 6–8 naive Br mosquitoes using a Nanoject II handheld injector (Drummond) as described by Dutra^26^. Each mosquito was injected intrathoracically with 207 nL of saliva. Injected mosquitoes were collected at 5 days post-injection and stored at −80 °C.

### Direct detection of MAYV in saliva

For direct detection of MAYV in saliva samples, we used samples collected from Br and *w*Mel mosquitoes at 28 days post-infection (dpi) according to the methodology described above. MAYV levels in mosquito saliva (fresh and frozen virus) were quantified via Real Time qPCR (RT-qPCR) and primers specific for MAYV were used in a multiplex assay (see below). To improve detection, saliva samples were grouped into pools of two, forming 10 pairs for each group.

### MAYV quantification and *Wolbachia* detection

MAYV levels in orally infected *Ae*. *aegypti* mosquitoes were quantified using Real Time qPCR (RT-qPCR) using a LightCycler® 96 (Roche). A multiplex assay was performed with previously developed primers specific for MAYV: MayV-F *5*′*/GTGGTCGCACAGTGAATCTTTC/3*′/MayV-R *5′/CAAATGTCCACCAGGCGAAG/3* and May-Probe *5′/FAM/ATG GTG GTA GGC TAT CCG ACA GGT* C/3lABkFQ/3′^7^. The *Ae*. *aegypti* ribosomal S17 (RPS17) primers are 17S-F *5′/TCC GTG GTA TCT CCA TCA AGC T/3*′/17S-R *5′/CAC TTC CGG CAC GTA GTT GTC/3′* and probe 5′/HEX/*CAG GAG GAG GAA CGT GAG CGC AG*/3BHQ2/3′^20^. The primers for *Wolbachia* detection in cells and mosquito samples were WSP-TM2 F: 5′-CAT TGG TGT TGG TGT TGG TG-3′/WSP-TM2 R: 5′-ACA CCA GCT TTT ACT TGA CCA G-3′ and probe 5′-/56-FAM/TCC TTT GGA/ZEN/ACC CGC TGT GAA TGA/3lAbRQSp/-3′^30^. All fluorophores were modified from those presented in the original publications for use in our multiplex assay.

Total RNA from the mosquito heads + thoraces was extracted with the High Pure Viral Nucleic Acid Kit (Roche) following the manufacturer’s instructions. RNA samples were quantified using a Thermo Scientific™ NanoDrop 2000, diluted to 50 ng/µL in nuclease-free water, and stored at −80 °C.

Thermocycling conditions were as follows: an initial reverse transcription step at 50 °C for 10 min; RT inactivation/initial denaturation at 95 °C for 30 s, and 40 cycles of 95 °C for 5 s and 60 °C for 30 s, followed by cooling at 37 °C for 30 s. The total reaction volume contained 10 µL (5 × LightCycler^®^ Multiplex RNA Virus Master (Roche), 1 µM primers and probe, and 125 ng of RNA template).

All samples were tested in duplicate for MAYV, WSP-TM2 and RPS17 and were analyzed using absolute quantification through serial dilutions of cloned target gene product into pGEMT-Easy plasmid (Promega) according to the manufacturer’s instructions. A negative control sample was normalized and used to determine a minimum threshold for positive samples. Absolute MAYV and WSP-TM2 copy numbers were calculated as the total number of copies per tissue or saliva sample.

### Data analysis

The data were first analyzed with the D’Agostino and Person omnibus normality test. Fisher’s exact test was then used to assess differences in viral prevalence. Viral load data were compared using a Mann-Whitney U test. Comparisons were considered to be significant for *P* values lower than 0.05. All analyses were performed using Prism V6 (Graphpad).

## Electronic supplementary material

Supplementary Figure S1
