# Supplementary material for: Wolbachia significantly impacts the vector competence of Aedes aegypti for Mayaro virus
Source: Sci Rep. 2018 May 2;8:6889. doi: 10.1038/s41598-018-25236-8 (PMC5932050; doi:10.1038/s41598-018-25236-8)
Supplement: Supplementary file 1 — Supplementary Figure S1 [file 41598_2018_25236_MOESM1_ESM.docx]

# SREP-17-53447

# *Wolbachia* significantly impacts the vector competence of *Aedes aegypti* for Mayaro virus

# Thiago Nunes Pereira^1,+^, Marcele Neves Rocha^1,+^, Pedro Henrique Ferreira Sucupira^1^, Fabiano Duarte Carvalho^1^, and Luciano Andrade Moreira^1*^.

**Supplementary files**

**Figure S1**.


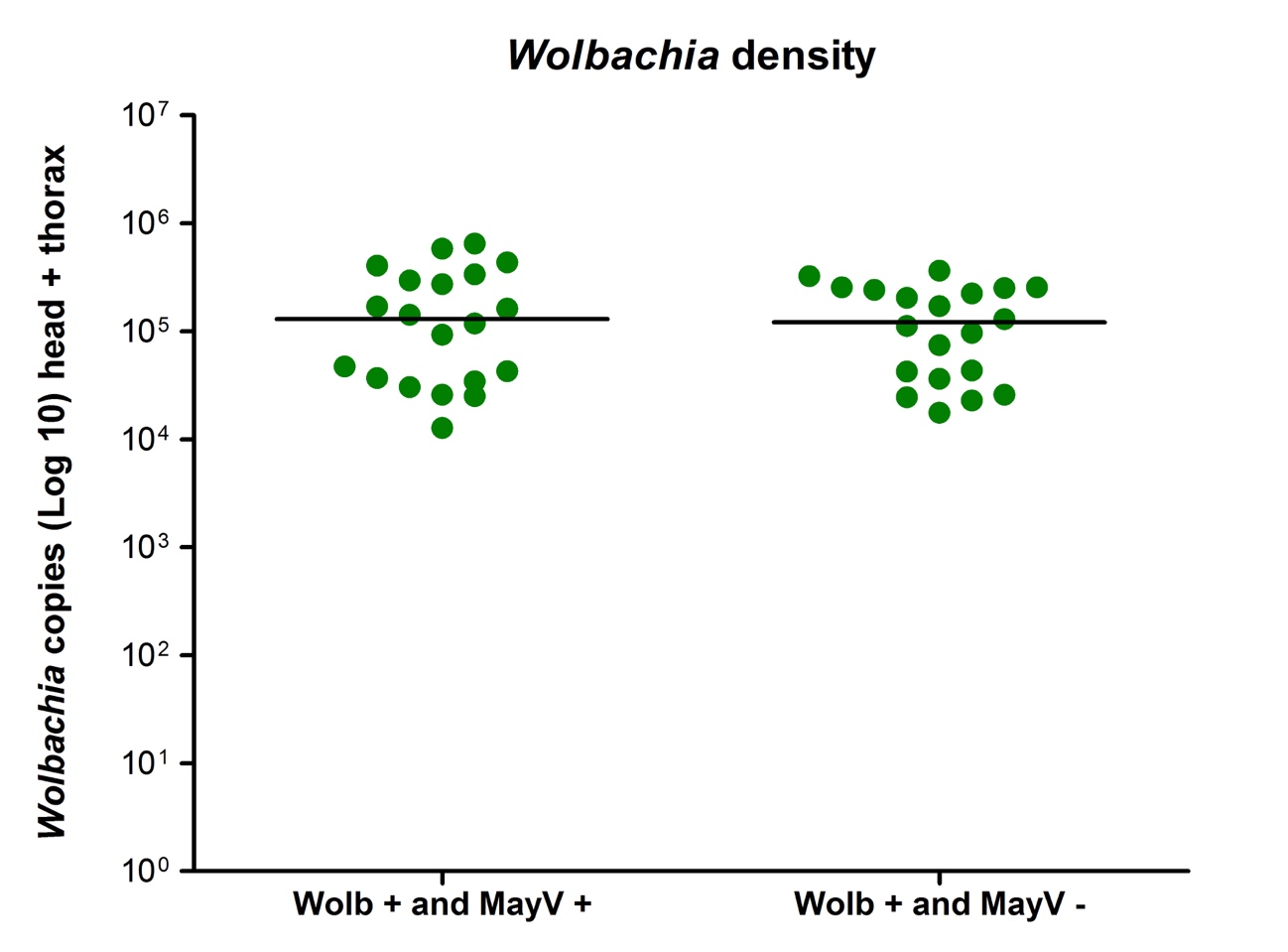


**Figure S1**. *Wolbachia* density in MAYV-positive and -negative mosquito samples.

Graph shows the *Wolbachia* density, based on a standard curve, on mosquito samples that were positive for MAYV (on the left) and MAYV-negative samples. Statistical analysis shows no significant difference on *Wolbachia* density between the two groups (Mann-Whitney U test, P=0.6349).
